# ARHGAP21 enhances metastasis in hepatocellular carcinoma by inhibiting ubiquitination of filamin A

**DOI:** 10.1038/s41420-026-03103-0

**Published:** 2026-04-09

**Authors:** Huijun Yao, Ziping Xie, Xingyu Tao, Xingyi Zhong, Xiaoxiao Wang, Kaiwen Xi, Zhiqin Zhu, Yangfeng Zhang, Feiye Liu, Junhao Lin, Fengsheng Chen

**Affiliations:** 1 Southern Medical University Hospital of Integrated Traditional Chinese and Western Medicine, Southern Medical University, Guangzhou, China; 2 Xiamen Branch, Zhongshan hospital, Fudan University, Shanghai, China; 3 Department of Clinical Research, State Key Laboratory of Oncology in South China, Guangdong Key Laboratory of Nasopharyngeal Carcinoma Diagnosis and Therapy, Guangdong Provincial Clinical Research Center for Cancer, Sun Yat-sen University Cancer Center, Guangzhou, P. R. China; 4 Department of Respiratory Medicine, National Key Clinical Specialty, Branch of National Clinical Research Center for Respiratory Disease, Xiangya Hospital, Central South University, Changsha, China; 5 Department of Medical Oncology, The People’s Hospital of Hezhou, Hezhou City, China; 6 Oncology Center, The Sixth Affiliated Hospital, School of Medicine, South China University of Technology, Guangzhou, China

**Keywords:** Cancer, Tumour biomarkers

## Abstract

Rho GTPase-activating protein 21 (ARHGAP21) plays a role in the occurrence and development of certain cancers, but its function in hepatocellular carcinoma (HCC) remains unclear. In this study, elevated ARHGAP21 expression was observed in both HCC cell lines and tissues and correlated with poor patient prognosis. Knockdown of ARHGAP21 suppressed HCC cell migration and invasion in vitro by regulating the actin cytoskeleton, while overexpression of ARHGAP21 had the opposite effect. In vivo, knockdown of ARHGAP21 inhibited HCC tumorigenesis and metastasis. Mechanistically, we demonstrated that ARHGAP21 directly binds to FLNA, and the PDZ domain of ARHGAP21 functions as a potential mediator of its binding to the 1–1200 aa fragment of FLNA. ARHGAP21 also directly binds to and recruits HSP90α to stabilize FLNA by inhibiting its ubiquitination and degradation. Overexpression of FLNA reversed the cytoskeleton remodeling-related suppression of tumor metastasis in ARHGAP21-knockdown HCC cells. These results revealed that ARHGAP21 promotes cytoskeleton remodeling and stimulates HCC metastasis by inhibiting FLNA ubiquitination and degradation via HSP90α recruitment. Our results position ARHGAP21 as both a potential prognostic marker and a promising therapeutic target in HCC.

## Introduction

Hepatocellular carcinoma (HCC) is the eighth most common malignancy worldwide, ranking third among all causes of cancer-related mortality [1]. The global incidence of HCC has increased in recent years, with a concerning rise in cases among younger individuals in particular regions [2]. Despite advancements in therapeutic strategies, including targeted therapies and immunotherapies [3–5], the prognosis for patients with advanced HCC accompanied by vascular invasion or extrahepatic spread remains dismal [6, 7]. Consequently, it is imperative to deepen the understanding of HCC pathogenesis and to identify novel, effective therapeutic targets to improve patient survival.

The Rho family GTPase RhoA regulates cell morphology and motility via cytoskeletal reorganization, thereby influencing migration and invasion [8, 9]. The nucleotide-bound state (GTP or GDP) and thus the activity of RhoA is precisely governed by guanine nucleotide-exchange factors (GEFs) and GTPase-activating proteins (GAPs): GEFs activate RhoA by catalyzing GDP release to enable GTP binding, whereas GAPs inactivate it by inducing hydrolysis of bound GTP to GDP [10, 11].

Rho GTPase Activating Protein 21 (ARHGAP21) contains a PDZ domain that mediates protein–protein interactions, a PH domain for membrane targeting, and a characteristic RhoGAP domain that negatively regulates RhoA and Cdc42 [12, 13]. ARHGAP21 is broadly expressed across multiple tissues, most notably in skeletal muscle and placenta, and exhibits a primary localization to the nucleus, perinuclear region, and cytoplasm. From these compartments, it can be recruited to the Golgi apparatus, cell junctions, or other sites to fulfill specific functional roles [12, 14]. Although ARHGAP21 has been implicated in contributing to the development and progression of tumors [15–17], the specific contribution of ARHGAP21 to HCC pathogenesis, its downstream effectors, and the molecular networks it engages remain largely unexplored.

In this study, we determined that ARHGAP21 expression is significantly upregulated in HCC tissues and cell lines, whereas knockdown of ARHGAP21 significantly suppresses the migration and invasion of HCC cells via cytoskeleton remodeling. Mechanistically, we identified a novel regulatory axis wherein ARHGAP21 recruits HSP90α to stabilize filamin A (FLNA) by inhibiting its ubiquitination and degradation. Therefore, these findings suggest that ARHGAP21 can function as a prognostic marker and that targeting of ARHGAP21 presents a potential therapeutic strategy for advanced HCC patients.

## Results

### ARHGAP21 expression as a biomarker of poor prognosis in HCC

**Fig. 1:**
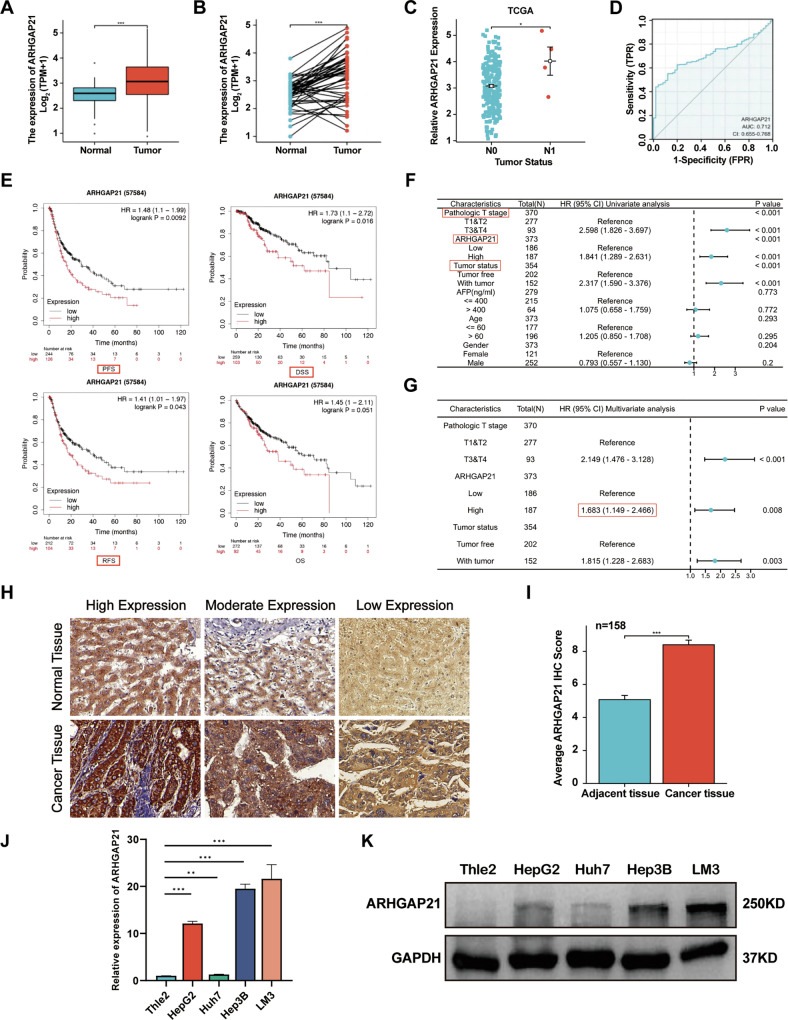
ARHGAP21 expression as a biomarker of poor prognosis in HCC. **A**, **B** ARHGAP21 expression levels were analyzed in the TCGA liver cancer dataset. Consistent upregulation of ARHGAP21 was observed throughout the study cohort, evident both in a comparison of 374 HCC samples against 50 normal liver tissue samples (**A**) and in 50 paired specimens (**B**). **C** Scatter plot representing ARHGAP21 expression across the TCGA cohort derived from tumors of 263 HCC patients. N0: no lymph node metastasis; N1: lymph node metastasis. Data displayed as mean ± SEM. *P < 0.05. **D** ROC analysis of TCGA liver cancer data using R yielded an AUC of 0.712 for ARHGAP21. **E** Kaplan–Meier plots were created from a liver cancer database. PFS progression-free survival, RFS recurrence-free survival, DSS disease-specific survival, OS overall survival. **F** Univariate Cox regression analysis revealed significant hazard ratios for T3/T4 stage (HR = 2.598), high ARHGAP21 expression (HR = 1.841), and tumor-bearing status (HR = 2.317). *P* < 0.001. **G** Multivariable Cox proportional hazards regression analysis of pathologic T stages, ARHGAP21 expression levels, and tumor status. **H**, **I** Characteristic immunoreactivity patterns (**H**) and quantitative scoring results (**I**) of ARHGAP21 expression in 158 pairs of HCC and paracarcinoma tissues. **J**, **K** Transcriptomic (qPCR, **J**) and proteomic (western blot, **K**) profiling of ARHGAP21 expression across HCC cell lines versus normal hepatic epithelial cell lines.

The initial objective of this study was to investigate the potential role of ARHGAP21 in HCC pathogenesis. Pan-cancer analysis of TCGA data revealed significantly elevated ARHGAP21 expression specifically in HCC (Fig. S1A). Subsequent analyses of mRNA expression datasets from the TCGA Liver Hepatocellular Carcinoma (LIHC) collection confirmed differential upregulation of ARHGAP21 in HCC tissues compared to non-tumor controls. This elevation was observed in both unpaired cohorts and matched tumor-normal pairs (Fig. 1A, B).

We next evaluated the clinical relevance of ARHGAP21 overexpression. In 263 TCGA HCC specimens, high ARHGAP21 expression correlated with lymph node metastasis (N + ) status, demonstrating significantly higher expression in N+ tumors versus N0 cases (Fig. 1C). A receiver operating characteristic (ROC) curve analysis using TCGA data indicated the diagnostic utility of ARHGAP21 for HCC (AUC = 0.712) (Fig. 1D). Importantly, a Kaplan–Meier analysis of the HCC cohort revealed significantly reduced progression-free survival, recurrence-free survival, and disease-specific survival in patients with high ARHGAP21 expression (Fig. 1E). Univariate Cox regression identified significant hazard ratios for T3/T4 stage (HR = 2.598), high ARHGAP21 expression (HR = 1.841), and residual tumor presence (HR = 2.317). A subsequent multivariate analysis confirmed ARHGAP21 as an independent prognostic factor for poor survival (HR = 1.683, 95% CI:1.149–2.466; *P* = 0.008) (Fig. 1F, G).

To validate these findings experimentally, we assessed ARHGAP21 expression in liver tissue microarrays by immunohistochemistry (IHC). ARHGAP21 levels were significantly elevated in the tumor tissue compared with those in the adjacent tissue (*P* < 0.001) (Fig. 1H, I). Analysis of 79 clinical HCC specimens further demonstrated an association of high ARHGAP21 expression with advanced T stage (Table S1), suggesting its prognostic relevance in disease progression. Finally, comparative assessment of basal ARHGAP21 expression demonstrated significant upregulation at both mRNA and protein levels in HCC cell lines relative to immortalized normal hepatocytes (Fig. 1J, K).

### ARHGAP21 promotes migratory and invasive phenotypes in HCC in vitro models through actin cytoskeleton regulation

**Fig. 2:**
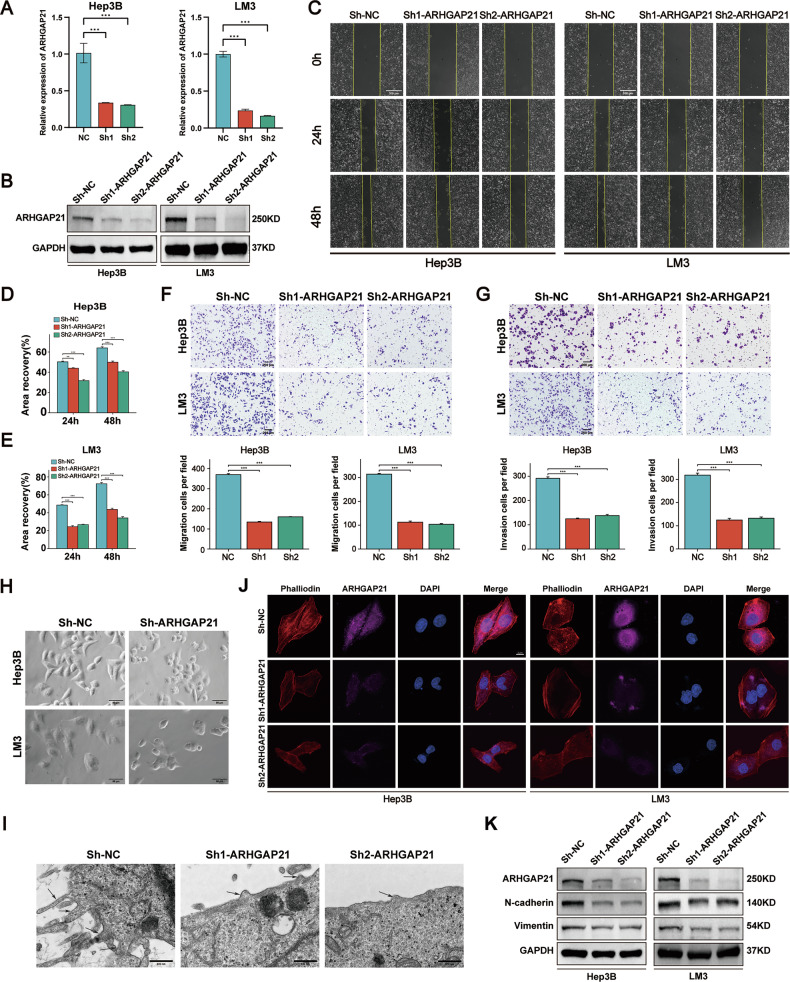
ARHGAP21 promotes migratory and invasive phenotypes in HCC in vitro models through actin cytoskeleton regulation. **A** The efficiency of stable ARHGAP21 knockdown by shRNA was evaluated by qRT-PCR. **B** ARHGAP21 stable knockdown was validated by western blot analysis. **C**‒**E** In vitro scratch assay showing characteristic migration fronts and kinematically analyzed displacement velocities. Scale bar, 300 μm. ***P < 0.001. **F**, **G** Representative Transwell images depict migratory and invasive capacities of relevant cell lines with corresponding statistical analysis. Scale bar, 200 μm. ***P < 0.001. **H** Phase-contrast micrographs comparing morphological alterations between wild-type and ARHGAP21-knockdown hepatocellular carcinoma cells. **I** Electron microscopic analysis of pseudopodial density and protrusion length. Scale bar, 500 nm. **J** Confocal microscopy (×63) compared F-actin organization in control and ARHGAP21-knockdown HCC cells. Scale bar, 2 μm. **K** Western blot analysis of epithelial-mesenchymal transition markers.

To investigate the biological functions of ARHGAP21, Hep3B and LM3 HCC cells were subjected to knockdown of ARHGAP21 expression via shRNA-encoding lentiviral transduction, with knockdown efficiency validated by quantitative real-time PCR and Western blot assays (Fig. 2A, B and Fig. S1B). The functional consequences of ARHGAP21 depletion on cell migration and invasion were quantified using scratch wound, Transwell migration, and Boyden invasion assays (Fig. 2C‒G).

Notably, ARHGAP21-depleted cells displayed contraction of the cell periphery and diminished pseudopodia formation (Fig. 2H). This finding was further corroborated by electron microscopy (Fig. 2I). These data suggest that ARHGAP21 plays a role in regulating actin cytoskeletal remodeling, as pseudopodia are actin-driven structures [18]. To mechanistically interrogate this phenotype, actin polymerization dynamics were visualized in knockdown cells using TRITC-phalloidin staining. ARHGAP21 deficiency induced profound disruption of actin filament organization, characterized by fragmented filaments and significantly suppressed pseudopodial formation (Fig. 2J). Immunoblotting analyses of epithelial-mesenchymal transition (EMT) markers revealed downregulated protein expression of N-cadherin and vimentin upon ARHGAP21 knockdown (Fig. 2K and Fig. S1C).

To establish reciprocity and definitively confirm the oncogenic function of ARHGAP21, Hep3B and LM3 cells were engineered to overexpress ARHGAP21 via lentiviral infection. Successful transduction was verified by immunoblotting (Fig. S2A). Consistent with a gain-of-function effect, ARHGAP21 upregulation potently enhanced motile behavior. Specifically, Transwell migration and Boyden chamber invasion assays documented significantly augmented migratory and invasive capacities (Fig. S2B, C). Immunoblot profiling of EMT markers confirmed upregulated protein expression of N-cadherin and vimentin in ARHGAP21-overexpressing cells (Fig. S2D). Corroborating the pro-motility phenotype, TRITC-phalloidin staining revealed robust pseudopodia extension and prominent stress fiber alignment in ARHGAP21-overexpressing cells (Fig. S2E).

### ARHGAP21 knockdown suppresses HCC tumorigenesis and metastasis in vivo

**Fig. 3:**
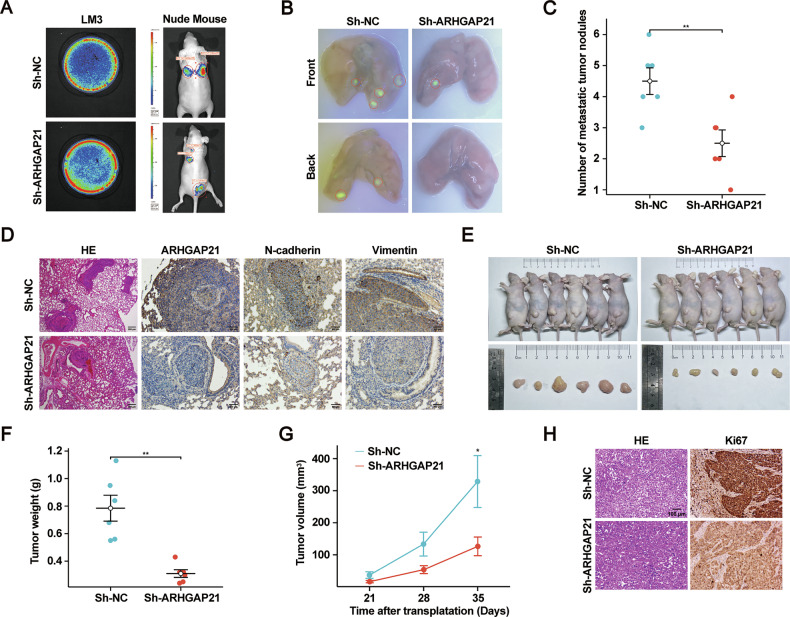
ARHGAP21 knockdown suppresses HCC tumorigenesis and metastasis in vivo. **A** Bioluminescence imaging of pulmonary metastases following tail vein injection of luciferase/BSR-expressing LM3 hepatocellular carcinoma cells in nude mice. **B**, **C** Bioluminescence images (**B**) and statistics (**C**) of pulmonary metastatic nodules in ex vivo lung tissue sections from nude mice. Data shown as mean ± SEM. **P < 0.01. **D** Metastatic colonization in pulmonary tissue was verified through H&E histological analysis. IHC staining of ARHGAP21, N-cadherin, and vimentin. Scale bars: 100 μm, 300 μm. **E** Subcutaneous xenograft model in nude mice. **F** Tumor xenograft sections were processed for H&E histology and proliferative activity assessment via Ki67 immunostaining. **G**, **H** Subcutaneous tumor weights (**G**) and volumes (**H**). *P < 0.05, **P < 0.01.

To evaluate the role of ARHGAP21 in HCC malignant progression in vivo, nude mice received tail vein injections of LM3-LV-shARHGAP21 cells to assess metastatic potential. Seven weeks post-injection, the mice were anesthetized and administered D-luciferin sodium salt intraperitoneally. Strikingly, bioluminescence imaging revealed significantly lower lung metastatic signal intensity (represented by Region of Interest, ROI, values) in the ARHGAP21-knockdown group compared to the negative control group (Fig. 3A). This reduced metastatic signal was corroborated by subsequent dissection and ex vivo fluorescence microscopy, which confirmed fewer lung metastatic foci in the ARHGAP21-knockdown mice (Fig. 3B, C). H&E staining of lung sections verified the histopathological characteristics and morphology of these metastatic lesions (Fig. 3D). IHC analyses performed on these lung tumors for ARHGAP21 itself and for the EMT markers vimentin and N-cadherin demonstrated significantly reduced staining intensity in the shARHGAP21 experimental group compared to negative controls (Fig. 3D). To complement the metastasis findings and directly evaluate the impact of ARHGAP21 knockdown on primary tumor growth, an LM3 cell line stably expressing ARHGAP21 shRNA was transplanted into nude mice to generate subcutaneous xenograft tumors. A significant inhibitory effect was also observed in this model, as tumor growth was significantly inhibited in mice bearing ARHGAP21-knockdown xenografts relative to tumors in mice bearing control xenografts (Fig. 3E). Tumors derived from ARHGAP21-depleted cells exhibited significantly reduced weight and volume compared to control tumors (Fig. 3F, G). IHC for the proliferation marker Ki67 revealed significantly reduced proliferation rates within the ARHGAP21-knockdown tumors relative to controls (Fig. 3H). Taken together, these in vivo findings demonstrate that ARHGAP21 contributes to HCC tumorigenesis and metastatic spread.

### ARHGAP21 directly interacts with FLNA

**Fig. 4:**
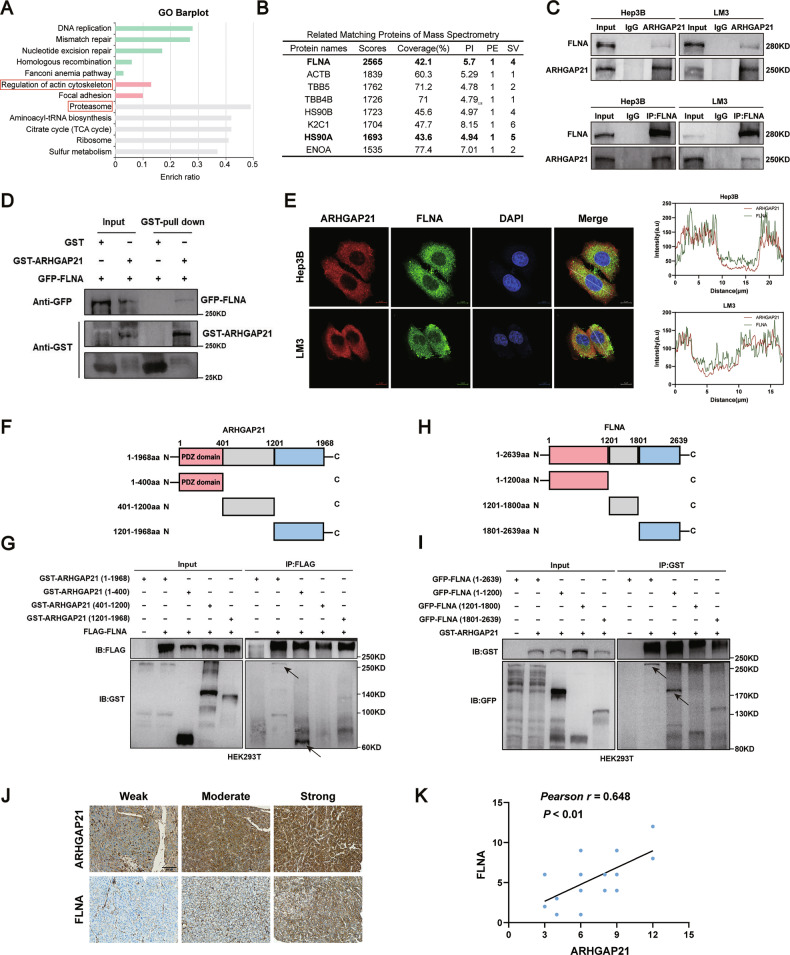
ARHGAP21 directly interacts with FLNA. **A** GO pathway. **B** The 8 most abundant proteins immunoprecipitating with ARHGAP21 from HCC cells, as identified by LC-MS/MS. **C** Binding between ARHGAP21 and FLNA was validated through reciprocal co-IP followed by western blotting. **D** GST pull-down assay demonstrating a physical interaction between GST-ARHGAP21 and GFP-FLNA. **E** Immunofluorescence images showing co-localization of ARHGAP21 (red) and FLNA (green). **F**, **G** Co-IP assays demonstrate an interaction between GST-tagged ARHGAP21 N-terminal fragment (residues 1–400) and wild-type FLNA in HEK293T cells. **H**, **I** Co-IP assays demonstrate an interaction between GFP-tagged FLNA N-terminal fragment (residues 1–1200) and wild-type ARHGAP21 in HEK293T cells. **J** IHC staining of ARHGAP21 and FLNA in an HCC tissue microarray from 17 patients. Scale bar, 300 µm. **K** Pearson’s correlation analysis.

We aimed to elucidate the regulatory mechanism of ARHGAP21 in HCC. We performed mass spectrometry (MS) on complexes isolated by immunoprecipitation (IP) of ARHGAP21 from HCC cells to identify its interacting partners. A GO pathway enrichment analysis conducted on proteins specifically isolated in ARHGAP21-containing complexes was consistent with an involvement of ARHGAP21 in regulating the actin cytoskeleton (Fig. 4A). Figure 4B shows the top eight scoring proteins in the IP/MS analysis; the cytoskeleton-related protein FLNA achieved the highest score. Co-IP assays demonstrated a physical interaction between ARHGAP21 and FLNA in HCC cells (Fig. 4C). This binding was further verified as direct using glutathione S-transferase (GST) pull-down assays with recombinant proteins (Fig. 4D). In addition, immunofluorescence staining indicated cytoplasmic co-localization of ARHGAP21 and FLNA within these cells (Fig. 4E).

To delineate the precise interaction domains between ARHGAP21 and FLNA, co-IP assays were performed using multiple truncated constructs of each protein. The results demonstrated that the N-terminal domain of ARHGAP21 (GST-ARHGAP21-1-400), which includes a PDZ domain [19], robustly interacts with wild-type (WT) FLNA protein (Fig. 4F, G). Similarly, the N-terminal domain of FLNA (GFP-FLNA-1-1200) co-precipitated with GST-tagged full-length ARHGAP21 (Fig. 4H, I). Given that PDZ domains are well-established mediators of protein–protein interactions that are often associated with cytoskeletal function [20, 21], these findings provide experimental evidence that supports the functional relevance of the PDZ domain of ARHGAP21 as a potential mediator of its binding to FLNA.

The interaction of ARHGAP21 with FLNA led us to investigate a potential role for FLNA in mediating the effects of ARHGAP21 on HCC development or progression. Interestingly, in an analysis of the TCGA-LIHC dataset, we found that FLNA mRNA expression was consistently and significantly higher in tumor tissue compared to normal tissue and was correlated with T stage (Fig. S3A, B), suggesting that the level of FLNA is related to cancer progression. We did not observe a significant difference in FLNA mRNA expression in ARHGAP21-depleted HCC cells relative to control cells (Fig. S3C). IHC staining was used to explore the correlation between ARHGAP21 and FLNA in a tissue microarray containing samples from 17 HCC patients. Pearson’s correlation analysis revealed that ARHGAP21 expression was positively correlated with FLNA expression in HCC patients (Fig. 4J, K). The positive correlation of FLNA with ARHGAP21 is consistent with a model in which FLNA plays a key role in ARHGAP21-mediated oncogenesis in HCC.

### ARHGAP21 and FLNA interact with HSP90α

**Fig. 5:**
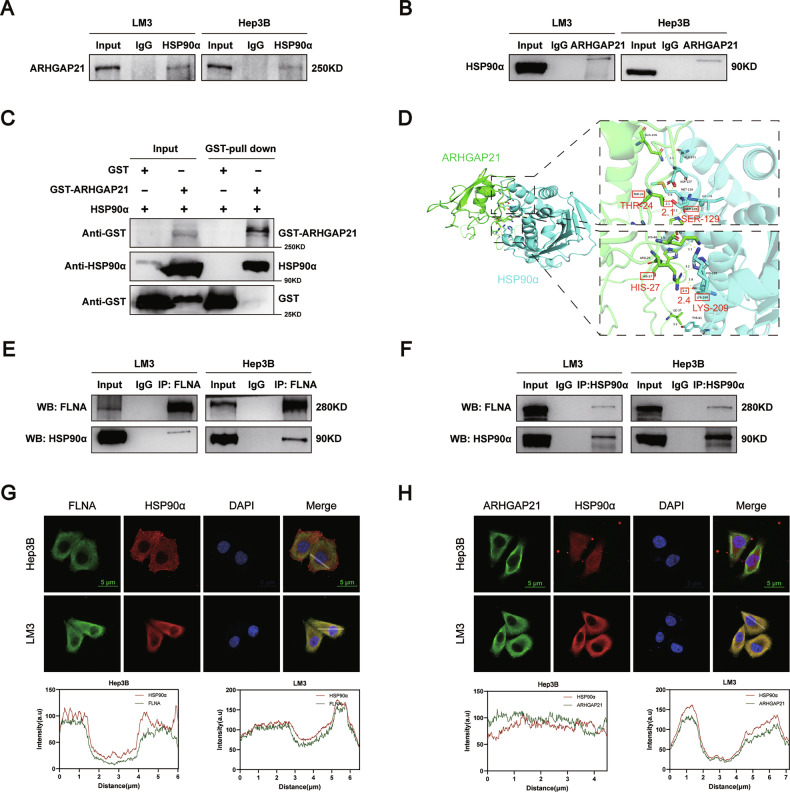
ARHGAP21 and FLNA interact with HSP90α. **A**, **B** Binding between ARHGAP21 and HSP90α was validated through reciprocal co-IP followed by western blotting. **C** GST pull-down assay. **D** Essential binding residues (cyan: HSP90α, green: ARHGAP21) appear in stick form. Yellow broken lines mark H-bonds with measured distances. **E**, **F** Co-IP followed by western blotting. **G**, **H** Immunofluorescence co-localization assay. Scale bar, 5 μm.

The MS analysis of potential ARHGAP21-binding proteins indicated an interaction of ARHGAP21 with HSP90α. HSP90α is a molecular chaperone that maintains the stability and function of cancer-associated client proteins [22, 23]. Co-IP assays confirmed interactions between ARHGAP21 and HSP90α in both Hep3B and LM3 HCC cells (Fig. 5A, B), and direct binding between ARHGAP21 and HSP90α was validated using GST pull-down assays (Fig. 5C). In silico protein–protein docking analyses were performed using the HDOCK server to investigate the potential interaction between ARHGAP21 and HSP90α. Notably, the hydrogen bond distances between Thr24 of ARHGAP21 and Ser129 of HSP90α, and between His27 of ARHGAP21 and Ser129 of HSP90α, were both less than 2.5 Å, indicating bond stability of the complex (Fig. 5D).

Interestingly, similarly, FLNA was also found to associate with HSP90α in these HCC cells (Fig. 5E, F), suggesting the potential existence of a ternary complex consisting of ARHGAP21, FLNA, and HSP90α. Indeed, confocal microscopy demonstrated cytoplasmic co-localization of ARHGAP21 and FLNA with HSP90α (Fig. 5G, H).

### ARHGAP21 recruits HSP90α to inhibit FLNA ubiquitination and proteasomal degradation in HCC

**Fig. 6:**
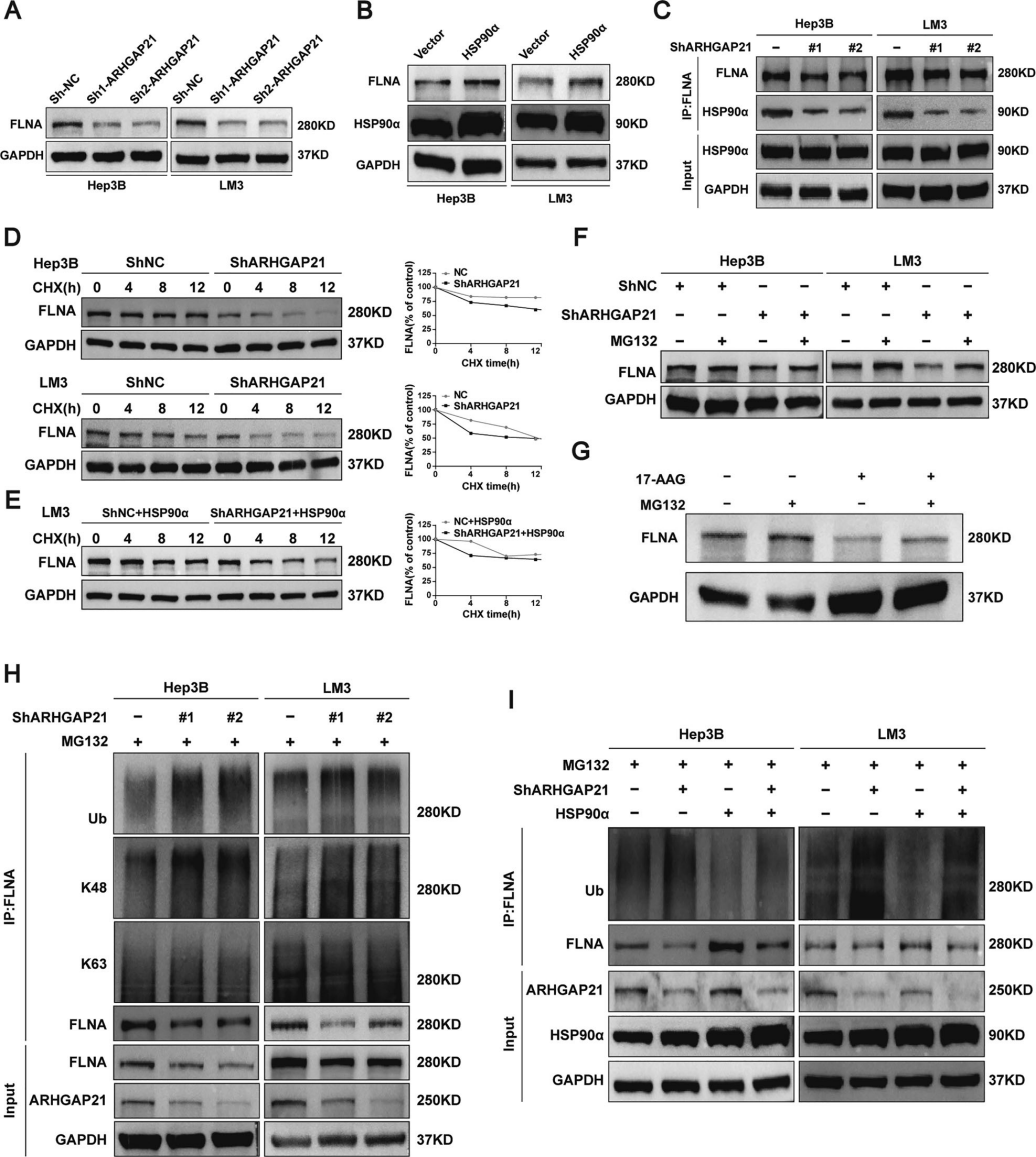
ARHGAP21 recruits HSP90α to inhibit FLNA ubiquitination and proteasomal degradation in HCC. **A** Western blot analysis detected FLNA protein levels in HCC cells in which ARHGAP21 was knocked down. **B** Western blotting of FLNA and HSP90α following transient HSP90α overexpression in HCC cells. **C** Immunoprecipitation determined the interaction of FLNA with HSP90α in ARHGAP21-knockdown HCC cells. **D** Cycloheximide (CHX) chase assay analyzing FLNA protein half-life in ARHGAP21-knockdown and control HCC cells treated with 100 μg/mL CHX. **E** CHX chase analysis detected the effects of HSP90α overexpression on protein stability of FLNA. **F** FLNA protein stability was evaluated in control and ARHGAP21- knockdown cells following treatment with either vehicle (DMSO) or MG132 (5 μM). **G** FLNA expression was analyzed by immunoblotting following treatment with 17-AAG (50 μM) or MG132 (50 μM) for 12 h. **H** FLNA was immunoprecipitated from ARHGAP21-deficient HCC cells, and western blotting was used to investigate K48- and K63-linked ubiquitination. **I** Co-IP/western blot measured FLNA ubiquitination changes upon ARHGAP21 knockdown and HSP90α overexpression in HCC cells.

We next investigated the functional consequences of the interactions among ARHGAP21, FLNA, and HSP90α in HCC. According to Western blotting analyses, FLNA levels were notably decreased in ARHGAP21-depleted HCC cells, suggesting that ARHGAP21 might play a role in stabilizing FLNA (Fig. 6A), but FLNA levels were significantly increased in HCC cells overexpressing HSP90α (Fig. 6B and Fig. S4A). Depletion of ARHGAP21 in HCC cells did not cause an obvious alteration to the expression of HSP90α, but ARHGAP21 depletion did reduce the interaction between HSP90α and FLNA (Fig. 6C and Fig. S4B). Cycloheximide chase assays demonstrated a significantly shortened FLNA half-life in ARHGAP21-knockdown cells compared to controls (Fig. 6D), and overexpression of HSP90α in these cells reversed the ARHGAP21-induced alteration in FLNA half-life (Fig. 6E).

These changes are consistent with a model in which ARHGAP21 stabilizes FLNA in a manner that involves recruitment of the molecular chaperone HSP90α. GO enrichment analysis of ARHGAP21-interacting proteins revealed significant enrichment in the proteasome pathway, suggesting that ARHGAP21 may increase levels of FLNA by stabilizing it against ubiquitin-dependent degradation (Fig. 4A). The downregulation of FLNA induced by ARHGAP21 knockdown was reversed by inhibition of the proteasome with MG132 treatment for 12 h (Fig. 6F). Similarly, while pharmacological inhibition of HSP90α with 17-AAG resulted in decreased FLNA levels, this effect was also blocked by MG132 (Fig. 6G).

IP followed by western blotting showed that ARHGAP21 knockdown promoted K48-linked, but not K63-linked, ubiquitination of FLNA (Fig. 6H and Fig. S4C). Notably, the extent of the effect of ARGHAP21 knockdown on K48-linked ubiquitination of FLNA was similar to the effect of the knockdown on total FLNA ubiquitination, suggesting that the K48 site is the dominant target of this effect. IP and Western blotting results further demonstrated that ARHGAP21 inhibits FLNA ubiquitination in an HSP90α-dependent manner (Fig. 6I). These results suggested that ARHGAP21 protects against K48-linked ubiquitination and subsequent degradation of FLNA by recruiting HSP90α.

### FLNA overexpression reverses the antitumor effect of ARHGAP21 deficiency in HCC Cells

**Fig. 7:**
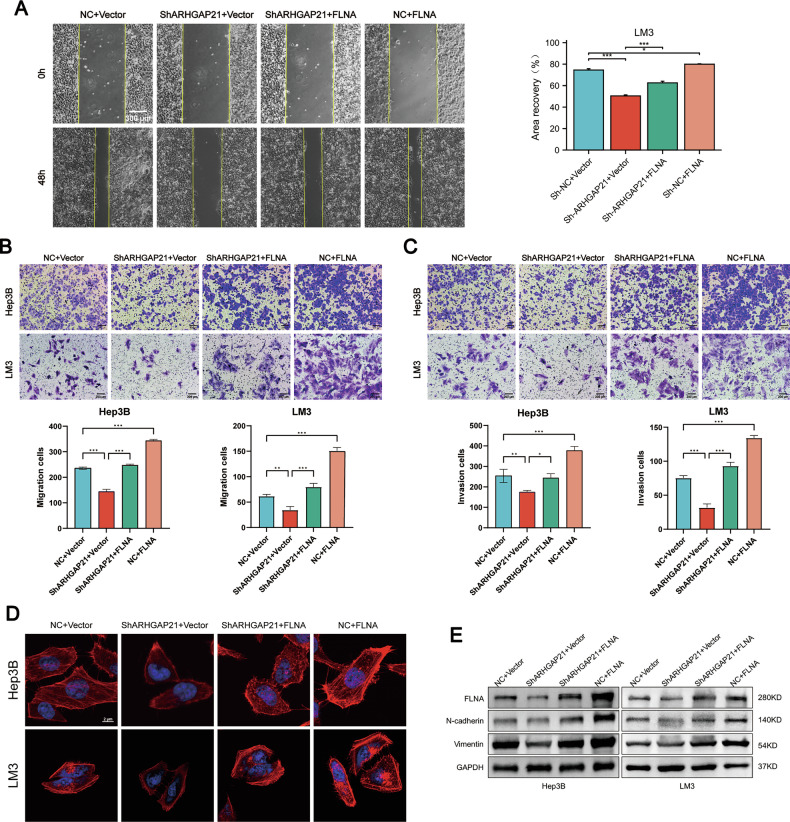
FLNA overexpression reverses the antitumor effect of ARHGAP21 deficiency in HCC cells. **A**–**C** Migration and invasion capabilities of FLNA-transfected HCC cells were tested through (**A**) scratch assays, **B**, **C** Transwell assays. *P < 0.05, **P < 0.01, ***P < 0.001. Scale bars: 200 μm, 300 μm. **D** FLNA was overexpressed in ARHGAP21-depleted Hep3B/LM3 cells, and F-actin was imaged by confocal microscopy (63×). Scale bar, 2 μm. **E** Western blot analysis of epithelial-mesenchymal transition markers.

To investigate the relationship between ARHGAP21 and FLNA, FLNA was overexpressed via plasmid transfection in HCC cell lines in which ARHGAP21 was stably knocked down. As demonstrated by scratch wound healing, Transwell migration, and Boyden chamber invasion assays, FLNA overexpression rescued the ARHGAP21 knockdown-induced suppression of migration and invasion in HCC cells (Fig. 7A–C). Furthermore, confocal microscopy analysis of TRITC-phalloidin-stained cells revealed that FLNA overexpression reversed the disruption of parallel actin filament structures and the inhibition of pseudopodia formation induced by ARHGAP21 knockdown (Fig. 7D and Fig. S4D). Western blotting analysis confirmed the increased level of FLNA and showed upregulated expression of N-cadherin and vimentin (Fig. 7E). Taken together, these results indicated that the promotion of HCC progression and metastasis occurs primarily through the impact of ARHGAP21 on FLNA.

## Discussion

HCC is characterized by aggressive tumor progression and a high propensity for both intrahepatic and extrahepatic metastasis, contributing to poor survival rates [7, 24]. Therefore, investigating the underlying mechanisms driving resistance and metastasis in HCC is critically important in order to support the development of more effective therapeutic strategies.

As a RhoGAP family member, ARHGAP21 negatively regulates Rho GTPase signaling, modulating actin cytoskeleton dynamics to influence cell migration and proliferation. The role of ARHGAP21 in cancer is context-dependent, demonstrating either oncogenic or tumor-suppressive activity in different cancer types. It has been reported that ARHGAP21 is overexpressed in cholangiocarcinoma and head and neck squamous carcinomas [17, 25]. Conversely, Bigarella et al. proposed that ARHGAP21 suppresses glioblastoma cell migration by inhibiting CDC42 via its RhoGAP domain, suggesting it has tumor suppressor functionality [13]. The results of Lazarini et al. [15] even suggested a simultaneous dual role for ARHGAP21, as silencing ARHGAP21 was found to both enhance cell migration and reduce proliferation in prostate adenocarcinoma cell lines. Despite the apparent importance of this protein in cancer, the expression profile and functional role of ARHGAP21 in HCC had remained largely unexplored.

In this study, we first identified the upregulation of ARHGAP21 in liver cancer through analysis of TCGA datasets and found that this upregulation is strongly associated with advanced T stage and lymph node metastasis. We further confirmed in tissue sections from liver cancer patients that ARHGAP21 expression is significantly elevated and positively correlated with advanced T stage. Our experimental evidence suggested a causal relationship underlying these connections, as knockdown of ARHGAP21 from cultured HCC cells potently suppressed migration and invasion. Interestingly, we observed that ARHGAP21 depletion in HCC cells impairs pseudopodium formation and alters cell morphology, both of which were associated with disrupted cytoskeletal dynamics. These data suggested that ARHGAP21 promotes the metastatic potential of HCC cells by inducing cytoskeleton remodeling. ARHGAP21 has also been shown to interact with α-tubulin and to influence acetylation of α-tubulin during EMT [26], thus potentially altering α-tubulin stability. This effect might cause the slight decrease in the levels of α-tubulin that was hinted at by slightly reduced phalloidin staining in ARHGAP21-knockdown cells (Fig. 2J). Our subsequent results revealed that knockdown of ARHGAP21 inhibits primary tumor metastasis in mouse models.

To investigate the molecular mechanisms through which ARHGAP21 promotes liver cancer metastasis, we screened for its interacting proteins in HCC cells using co-IP/MS. GO pathway enrichment analysis of these proteins confirmed significant enrichment of ARHGAP21 in actin cytoskeleton regulation. The cytoskeleton is crucial for controlling multiple factors important to metastasis, including cell shape, motility, transport, and environmental interactions [27, 28]. One actin-binding protein in particular, FLNA, was found to strongly interact with ARHGAP21. FLNA functions as an essential scaffold that cross-links and stabilizes actin networks [29–31]. FLNA orchestrates cell motility and invasion by regulating pseudopod formation through a process that involves cytoskeleton remodeling mediated by myosin and other proteins as well as components of focal adhesions [18, 32, 33]. Previous studies have demonstrated that FLNA serves as a prognostic biomarker, predicting early recurrence in post-hepatectomy HCC patients and associating with adverse clinical outcomes in pancreatic cancer [34, 35]. Using co-IP and pull-down assays, our study demonstrated that ARHGAP21 directly interacts with FLNA. In addition, consistent with previous research reporting that PDZ domains are involved in mediating protein–protein interactions [36], our study provided evidence that the PDZ domain of ARHGAP21 mediates its binding to FLNA.

HSP90α was another strong hit that was identified in our IP-MS analysis of ARHGAP21-binding proteins. We confirmed that ARHGAP21 and FLNA associate with and colocalize with HSP90α in the cytoplasm. Moreover, we observed that knockdown of ARHGAP21 significantly reduces FLNA protein levels in cells, but FLNA protein levels were upregulated following the overexpression of HSP90α. HSP90α has been shown to maintain the stability and function of other cancer-associated client proteins by suppressing their ubiquitination and subsequent degradation; this activity of HSP90α plays critical roles in the cancer-promoting activities of these client proteins [22, 37]. Therefore, our results support a model in which HSP90α is a critical mediator of the effects of ARHGAP21 on HCC cell metastasis, though such a direct involvement of HSP90a requires additional investigation. Similarly, while we have shown that mediation of the FLNA/HSP90α interaction is the primary driver of the pro-metastatic function of ARHGAP21, we cannot exclude minor contributions from other mechanisms, including those involving the protein’s canonical GAP activity.

Among polyubiquitin chain types, linkages via K48 and K63 are particularly abundant. K48-linked chains generally direct substrates to proteasomal degradation, and whereas K63-linked chains can serve as a foundation for generating K48/K63-branched ubiquitin chains, which also facilitate proteasomal degradation [38, 39]. Other ubiquitin chain types mediate distinct biological pathways: linear (M1) chains regulate NF-κB activation, K27-linked chains fine-tune innate immune signaling, and both K29- and K33-linked chains modulate the activity of various kinases [40–42]. Our results showed that ARHGAP21 protects FLNA from K48-linked ubiquitination and degradation in a manner dependent on recruitment of HSP90α.

**Fig. 8:**
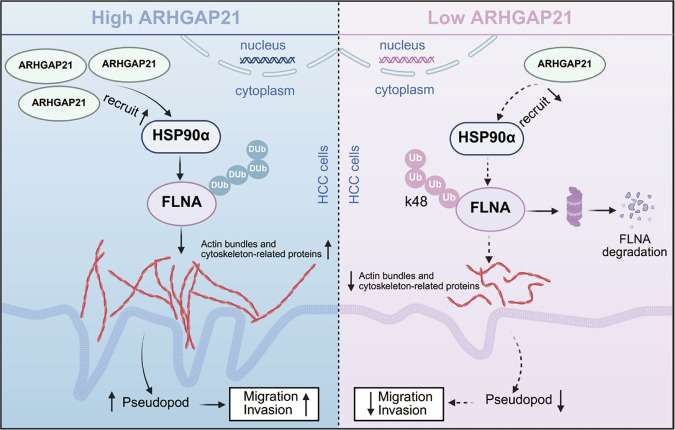
Schematic representation of the role of ARHGAP21 in HCC. ARHGAP21 recruits HSP90α to inhibit FLNA K48-linked ubiquitination and proteasomal degradation, thereby promoting cytoskeleton remodeling-stimulated HCC metastasis.

In summary, this study demonstrated for the first time that ARHGAP21 exhibits a pro-metastatic function in HCC. We identified ARHGAP21 upregulation in HCC, correlating with cytoskeletal remodeling, and established its critical role in driving invasion and metastasis. Molecular studies demonstrated that ARHGAP21 directly interacts with FLNA and HSP90α and prevents FLNA K48-linked ubiquitination and degradation through recruiting HSP90α, thereby promoting cytoskeleton remodeling-stimulated HCC metastasis (Fig. 8). These findings position ARHGAP21 expression as a prognostic marker in HCC and offer ARHGAP21 activity as an attractive potential target in the treatment of patients with advanced HCC.

## Materials and methods

### Public data analysis

Transcriptomic datasets from TCGA were analyzed to identify differentially expressed genes in HCC tissues versus adjacent normal tissues. Statistical analysis was performed using R (v3.5.0), with significance thresholds set at *P* < 0.05 for differential expression and |R | > 0.3 with *P* < 0.05 for correlation analyses.

### Cell culture

The study utilized four established hepatocellular carcinoma cell models (HepG2, Hep3B, Huh7, LM3) and a non-malignant hepatic control line (Thle2), all sourced from Zhong Qiao Xin Zhou Biotechnology (Shanghai, China). These cells were maintained in high-glucose Dulbecco’s modified Eagle medium (DMEM) supplemented with 10% fetal bovine serum (FBS) and 1% penicillin/streptomycin at 37 °C in a humidified incubator with 5% CO_2_. HEK293T cells were maintained in high-glucose DMEM supplemented with 10% fetal bovine serum (FBS), and cultured at 37 °C in a humidified 5% CO_2_ atmosphere. All cell lines were authenticated by STR profiling and routinely tested to ensure they were free of mycoplasma contamination.

### Transient transfection and lentivirus infection

HCC and 293T cells were plated at 30 to 50% confluence prior to transient transfection using Lipofectamine 3000 with P3000 reagent (Thermo Scientific, Waltham, MA, USA) to deliver siRNAs, plasmids, or truncation constructs. For stable genetic modification, Hep3B and LM3 cells were transduced with GFP-tagged ARHGAP21-overexpressing lentivirus (GeneChem, Shanghai, China) or ARHGAP21-knockdown lentiviral particles carrying shRNA (OBiO Technology, Shanghai, China) according to the manufacturers’ protocols. Puromycin selection was initiated at 72 h post-infection to establish stable pools. All constructs were sequence-verified, with overexpression or knockdown efficiency confirmed by GFP fluorescence and further validated through Western blot and qPCR analyses.

### RNA extraction and quantitative PCR

Total RNA was purified from cells using a commercial isolation kit (Foregene, China) as per the manufacturer’s guidelines. The extracted RNA was reverse transcribed into cDNA using the Evo M-MLV Mix Kit with gDNA Clean for qPCR (Accurate Biotechnology (Hunan)Co., Ltd., China). Quantitative real-time PCR was performed on a LightCycler480 II instrument (Roche, Switzerland) with SYBR Green Premix Pro Taq HS qPCR Kit (Accurate Biotechnology (Hunan) Co., Ltd., China). For normalization, β-actin served as the internal control, and relative gene expression was quantified via the 2^−ΔΔCt^ method.

### Wound-healing assay

Cells were inoculated into 6-well plates. After confluence was reached, the cultures were wounded by creating a scratch with a sterile pipette tip. Wound closure was monitored at 0, 24, and 48 h post-wounding using an Olympus microscope (Beijing, China). Migration rates were quantified by measuring the relative reduction in scratch width over time.

### Transwell assay

For migration assays, cells (5 × 10⁴ to 1 × 10⁵ per well) in serum-free DMEM were seeded into the upper chambers of Transwell systems (24-well format) with 8.0-µm pore polycarbonate inserts. The lower compartment contained DMEM supplemented with 10% FBS as a chemoattractant. For invasion assays, inserts were pre-coated with Matrigel (200 µg/mL; Corning, USA) for 1 h prior to seeding 5 × 10⁴ to 1 × 10⁵ cells in 100 µL serum-free DMEM in the upper chamber. Here, the lower chamber contained 500 µL DMEM/10% FBS. Following incubation at 37 °C for 18–24 h (migration) or 24 to 48 h (invasion), non-migrating/non-invading cells were removed from the upper membrane surface. Membranes were fixed with 4% paraformaldehyde for 15 min, stained with 0.1% crystal violet in methanol for 30 min, and rinsed with PBS. Transmigrated cells were quantified by counting three random microscopic fields per membrane insert at ×100 magnification, with results expressed as mean ± SD.

### Immunofluorescence

For immunofluorescence detection, cells were plated on glass-bottom confocal dishes and allowed to adhere. Cellular samples were fixed with 4% paraformaldehyde for 20 min at room temperature, then membrane permeabilization was accomplished by incubation with 0.5% Triton X-100 for 20 min. To delineate cytoskeletal organization, cells were incubated in the dark with 200 µL of prepared TRITC-conjugated phalloidin working solution (Beijing Solarbio Science & Technology Co., Ltd.) for 1 h at room temperature. For immunofluorescence co-localization studies, cells were sequentially stained with primary antibodies (ARHGAP21, FLNA, and HSP90α) overnight at 4 °C and subsequently incubated for 2 h at room temperature with appropriate fluorescent secondary antibodies. Fluorescence images were acquired using a Zeiss LSM800 confocal microscope (Carl Zeiss AG, Germany) with consistent imaging parameters across samples.

### Tail vein lung metastasis assay

LM3 cells stably transduced with sh-ARHGAP21 or a negative control (sh-NC) were further engineered to express firefly luciferase and blasticidin resistance (BSR) via lentiviral transduction, with successful transduction confirmed by BSR selection and in vitro bioluminescence imaging following D-luciferin sodium salt incubation. Twenty male nude mice (3 weeks old) were randomly assigned to two groups (*n* = 10 per group). Each mouse received a tail vein injection of LM3-luciferase cells (1.5 × 10⁶ in 100 µL PBS) transduced with either sh-ARHGAP21 or sh-NC. After 7 weeks, lung metastasis burden was quantified using in vivo bioluminescence imaging following intraperitoneal D-luciferin injection (150 mg/kg). Mice were then euthanized by cervical dislocation under anesthesia, and excised lungs were subjected to ex vivo fluorescence imaging for metastatic nodule enumeration, with subsequent histological analysis (H&E staining) for confirmation.

### Subcutaneous xenograft tumor model

LM3 cells stably transduced with ARHGAP21-knockdown lentivirus (sh3-ARHGAP21-LM3) or control virus (sh-NC-LM3) were used to create the subcutaneous tumorigenesis model. Sixteen 3-week-old male nude mice were randomly divided into two groups (*n* = 8 mice/group) and ear-punched for identification. Each mouse received a subcutaneous injection of 100 μL PBS containing 2 × 10^6^ cells. Palpable tumors became visible at the injection sites by day 21 post-inoculation. Tumor dimensions (length and width) were measured weekly using digital calipers, and tumor volume was calculated as 0.5× (length × width^2^). In accordance with ethical guidelines, the experiment was terminated when tumors reached approximately 1 cm in maximal diameter (day 35).

### IHC

The study utilized a commercially available liver cancer tissue array (HLivH180Su15; Shanghai Outdo Biotech, China), comprising tumor specimens and matched adjacent non-tumor tissues. The IHC evaluation was conducted using commercially available kits (Proteintech, Wuhan, China) for the detection of ARHGAP21, FLNA, vimentin, N-cadherin, and Ki67 protein expression. IHC evaluation was performed using established scoring criteria: samples with scores below 4 were considered negative, while those scoring 4 or higher were classified as positive. For tumor specimens specifically, expression levels were further stratified using a threshold of 6, with scores ≤6 representing low expression and scores >6 indicating high expression.

### Western blotting

Protein samples were separated electrophoretically using 7.5% precast polyacrylamide gels and transferred to PVDF membranes. Membranes were blocked for 1 h at 25 °C using 5% non-fat dry milk in TBST (0.05% Tween-20, 120 mM Tris-HCl pH 7.4, 150 mM NaCl). Following overnight incubation with primary antibodies at 4 °C (see Supplementary Table S2), membranes were washed three times with TBST and probed with HRP-conjugated secondary antibodies. Protein bands were visualized on a Minichemi imaging system (Sage Creation Science, Beijing, China).

### Co-IP

Protein–protein interactions were examined using the Pierce Co-Immunoprecipitation Kit (Thermo Fisher Scientific, USA) following the manufacturer’s recommended protocol. Immunoprecipitated complexes were subsequently resolved by SDS-PAGE and subjected to Western blot analysis for target protein detection.

### GST pull-down

GST pull-down assays were examined using a commercial GST pull-down kit (GeneCreate Biological Engineering, Wuhan) following the supplier’s recommended protocol. The assay was conducted with appropriate controls including GST-only and input lysate samples to verify specific interactions.

### Cycloheximide chase and ubiquitination assays

To assess protein turnover dynamics, cycloheximide (MedChemExpress, Monmouth Junction, NJ, USA) was administered to cells at 100 μg/mL for the indicated durations. For ubiquitination analysis, parallel cultures were pretreated with 5 μM MG132 (MedChemExpress, Monmouth Junction, NJ, USA) for 12 h prior to harvest. Cellular proteins were isolated with RIPA lysis buffer, heat-denatured in SDS-loading buffer (95 °C, 10 min), and subjected to Western blot analysis. For FLNA ubiquitination studies, FLNA-containing complexes were immunoprecipitated from IP lysis buffer extracts prior to electrophoretic separation.

### Statistical analysis

Survival outcomes (PFS, RFS, DFS, OS) were evaluated using Kaplan–Meier methodology or the Gene Expression Profiling Interactive Analysis platform. Associations between ARHGAP21 expression and clinicopathological parameters were examined through Fisher’s exact tests or χ² analyses. All quantitative data are reported as the mean ± standard deviation, derived from at least three separate experiments. For comparative analyses, Student’s *t* tests were employed for two-group comparisons, while one-way ANOVA was applied for multi-group assessments. All statistical computations were performed using GraphPad Prism 9.5 (GraphPad Software, San Diego, CA, USA), with two-tailed tests conducted throughout. Significance thresholds were established at *P* < 0.05, *P* < 0.01, or *P* < 0.001.

### Ethics approval

All animal studies were approved by Shenzhen Top Biotech Co., Ltd., Institutional Animal Care and Use Committee (ethical approval code: TOP-IACUC-2022-0230), and all methods were performed in accordance with the relevant guidelines and regulations.

## Supplementary information

Supplementary Information.

Original Data.

Raw Mass Spectrometry Data.

## Data Availability

All relevant data are available from the authors.
