# Supplementary material for: ARHGAP21 enhances metastasis in hepatocellular carcinoma by inhibiting ubiquitination of filamin A
Source: Cell Death Discov. 2026 Apr 9;12:240. doi: 10.1038/s41420-026-03103-0 (PMC13187493; doi:10.1038/s41420-026-03103-0)
Supplement: Supplementary file 1 — Supplementary Information. [file 41420_2026_3103_MOESM1_ESM.docx]

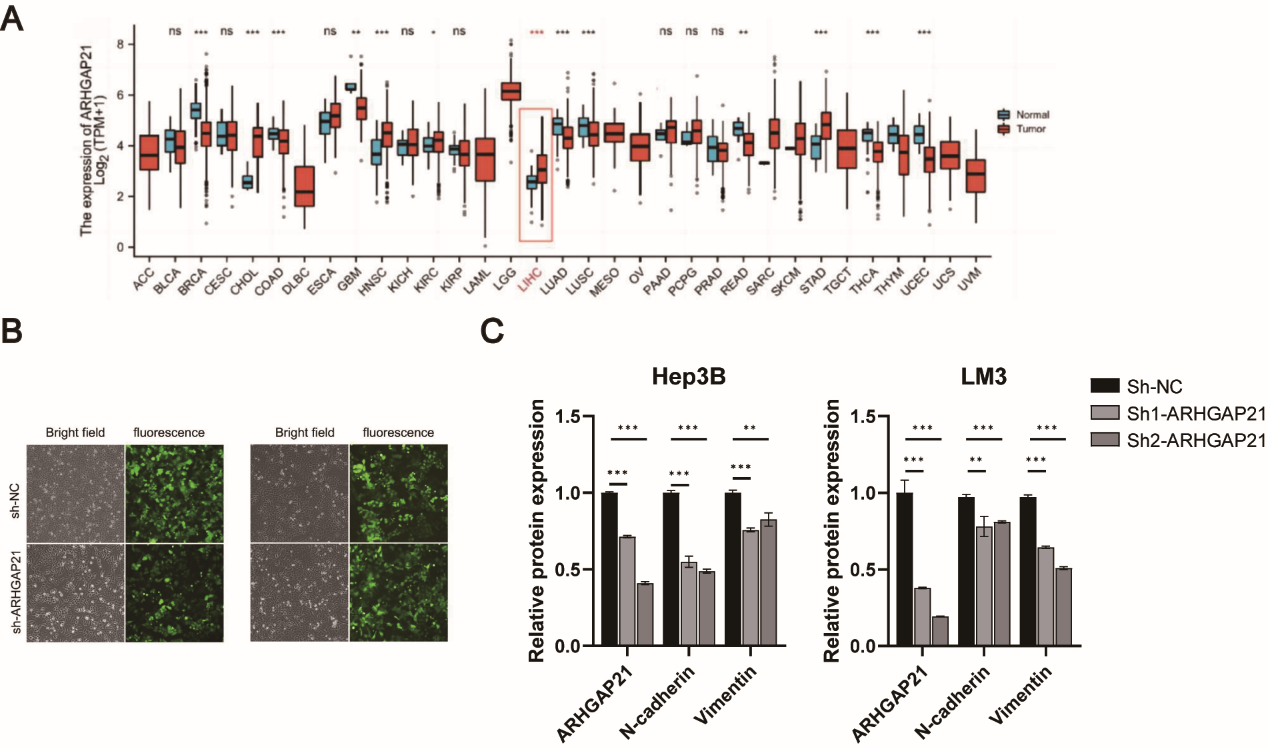


**Figure S1.**

(A) Analysis of TCGA pan-cancer data revealed that ARHGAP21 is significantly overexpressed in hepatocellular carcinoma (HCC) compared to other cancers (****p*<0.001).

(B) Lentiviral transduction of Hep3B and LM3 cells was confirmed by GFP expression.

(C) Quantification.


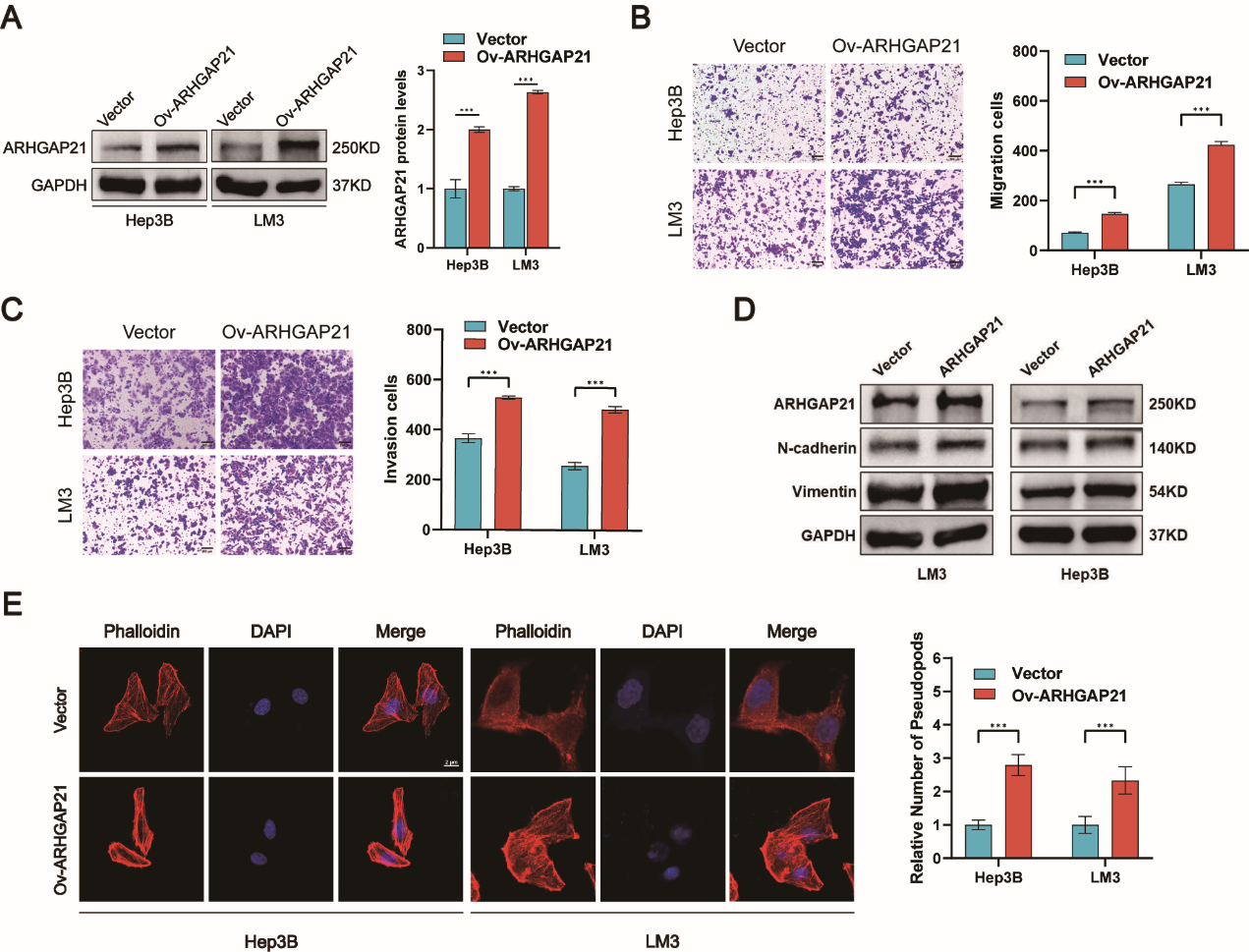


**Figure S2.**

(A) ARHGAP21 stable overexpress efficiency was validated by Western blot. ****p*<0.001.

(B) Transwell assays. Scale bar, 200 um. ****p*<0.001.

(C) Boyden assays. Scale bar, 200 um. ****p*<0.001.

(D) WB detection of EMT related markers.

(E) F-actin organization in control and ARHGAP21-overexpressing HCC cells by confocal microscopy (63×). Scale bar, 2um.


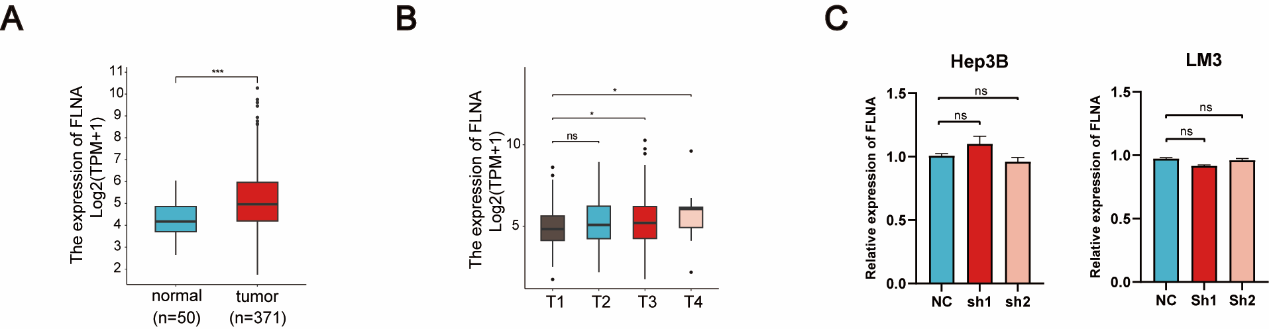


**Figure S3.**

(A) FLNA expression levels were analyzed in the TCGA liver cancer dataset

(B) T stage.

(C) The transcript levels of FLNA mRNA were measured by qRT-PCR. ns: not significant.


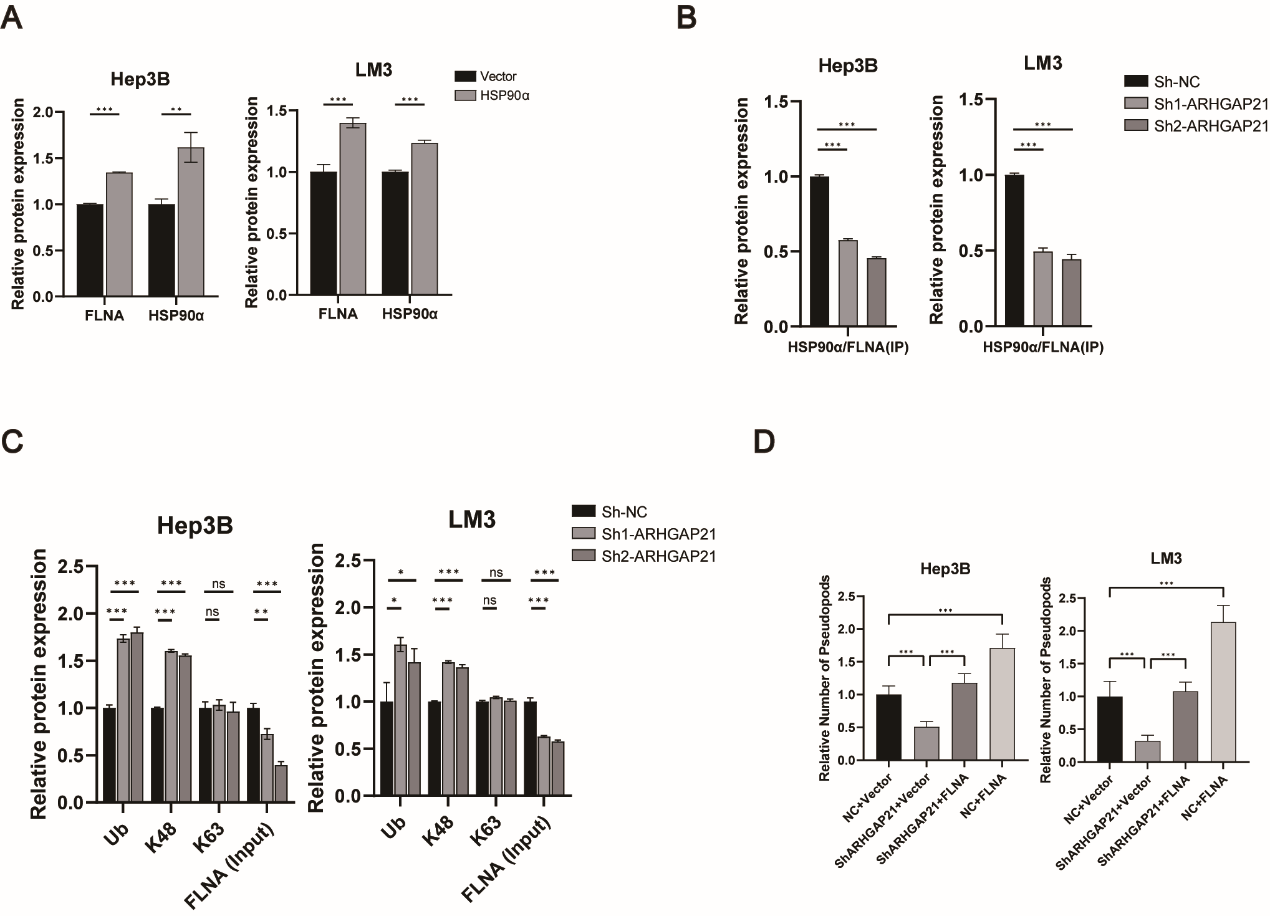


**Figure S4.**

1. Quantification of HSP90α overexpression in HCC cells. ****p*<0.001.
2. Quantification of the interaction between HSP90α and FLNA. ****p*<0.001.

(C) Quantification of total, K48-linked, and K63-linked ubiquitination on FLNA by immunoprecipitation in HCC cells. **p* < 0.05, ***p* < 0.01, ****p* < 0.001.

(D) Bar graph showing the relative number of pseudopods. ****p*<0.001.

| **Supplementary Table 1** Correlation between ARHGAP21 expression in tumor tissues and clinicopathological features of patients with liver cancer | | | | |
| --- | --- | --- | --- | --- |
|  |  | **ARHGAP21 expression** | |  |
| **Variables** | **N** | **low** | **high** | ***p*-value** |
| **Gende(%)** |  |  |  | 0.846 |
| **Female** | 6 | 3(3.8%) | 3(3.8%) |  |
| **Male** | 73 | 27(34.2%) | 46(58.2%) |  |
| **Age, mean ± sd** | 79 | 52.8 ± 10.813 | 52.388 ± 10.857 | 0.870 |
| **T stage(%)** |  |  |  | **< 0.001** |
| **T1** | 48 | 30(38%) | 18(22.8%) |  |
| **T2** | 27 | 0(0%) | 27(34.2%) |  |
| **T3** | 4 | 0(0%) | 4(5.1%） |  |
| **HbsAg(%)** |  |  |  | 0.021 |
| **Positive** | 18 | 11(13.9%) | 7(8.9%) |  |
| **Negative** | 61 | 19(24.1%) | 42(53.2%) |  |
| **Cirrhosis(%)** |  |  |  | 0.679 |
| **Positive** | 71 | 28(35.4%) | 43(54.4%) |  |
| **Negative** | 8 | 2(2.5%) | 6(7.6%) |  |
| **Tumor size in cm, median (IQR)** | 79 | 3(2.525, 6) | 4.5(3,7) | 0.475 |
| Chi-square test or Fisher’s exact test was applied to access the associations between the expression of ARHGAP21 and the clinicopathologic characteristics. | | | | |

| **Supplementary Table 2** The antibodies used in this study. | | |  |  |
| --- | --- | --- | --- | --- |
| Antibody name | Brand name | Cat: | Species | Dilution |
| ARHGAP21 | Proteintech | 22183-1-AP | Rabbit | WB: 1:1000; IHC: 1:50; IF: 1:50 |
| FLNA | Santa Cruz Biotechnology | sc-17749 | Mouse | WB: 1:1000; IHC: 1:50; IF: 1:50 |
| HSP90α | Abcam | ab128483 | Mouse | WB: 1:1000; IF: 1:50 |
| Vimentin | Proteintech | 10366-1-AP | Mouse | WB: 1:2000; IHC: 1:2500 |
| N-cadherin | Proteintech | 66219-1-Ig | Mouse | WB: 1:5000; IHC: 1:7500 |
| Ki67 | Proteintech | 27309-1-AP | Rabbit | IHC: 1:2000 |
| K48-linkage Specific Polyubiquitin | Selleck | F0527 (13) | Rabbit | WB: 1:1000 |
| K63-linkage Specific Polyubiquitin | Selleck | F0528 (16) | Rabbit | WB: 1:1000 |
| ubiquitin | Proteintech | 10201-2-AP | Rabbit | WB: 1:1500 |
| GST-tag | Cell Signaling Technology | 2525T | Rabbit | WB: 1:1000 |
| Flag-tag | Sigma | F1804 | Mouse | WB: 1:1000 |
| GFP-tag | Abcam | ab290 | Rabbit | WB: 1:1000 |
| GAPDH | Proteintech | 60004-1-Ig | Mouse | WB: 1:100000 |
