# Supplementary figures and images for: ARHGAP21 enhances metastasis in hepatocellular carcinoma by inhibiting ubiquitination of filamin A
Source: Cell Death Discov. 2026 Apr 9;12:240. doi: 10.1038/s41420-026-03103-0 (PMC13187493; doi:10.1038/s41420-026-03103-0)

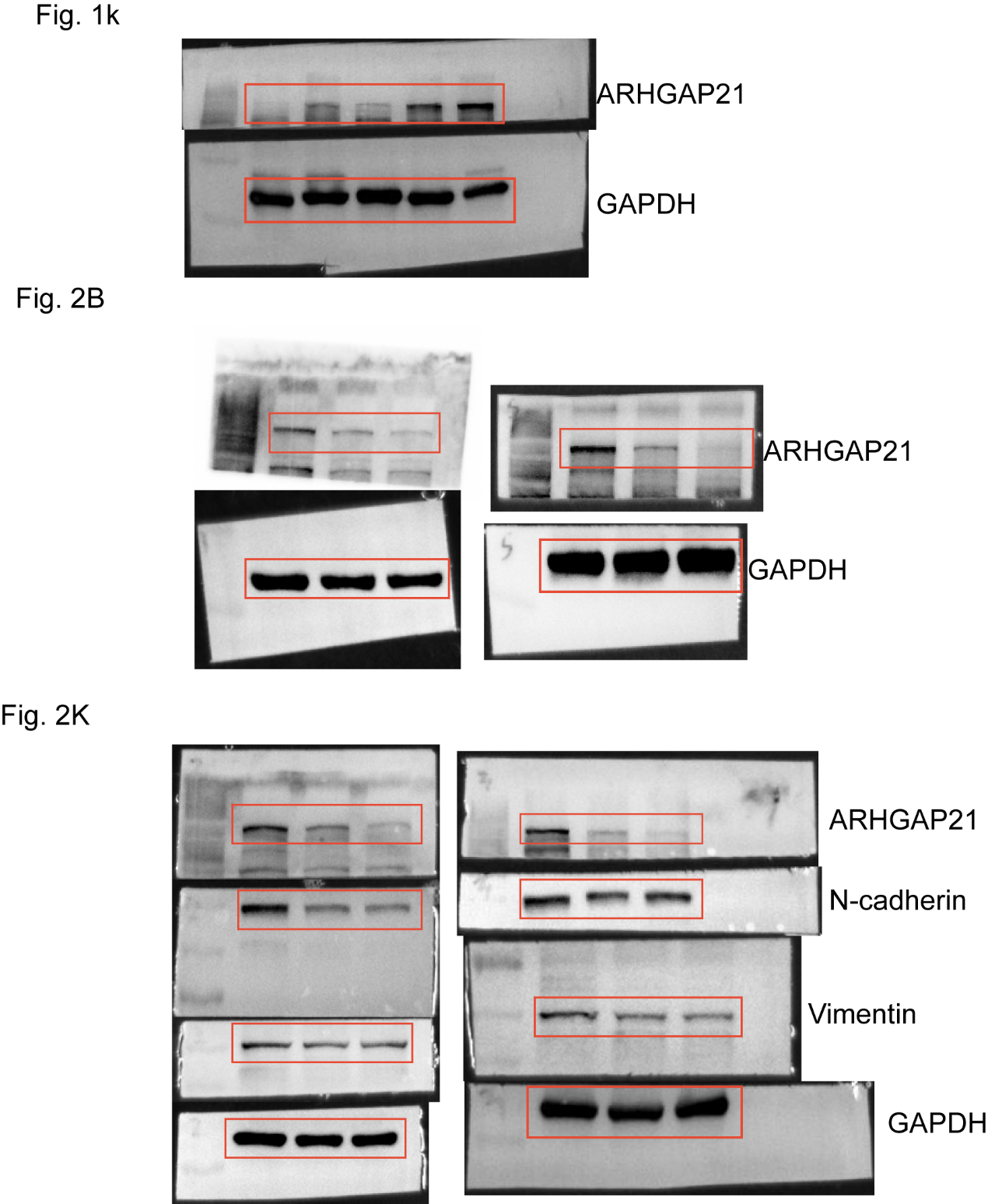


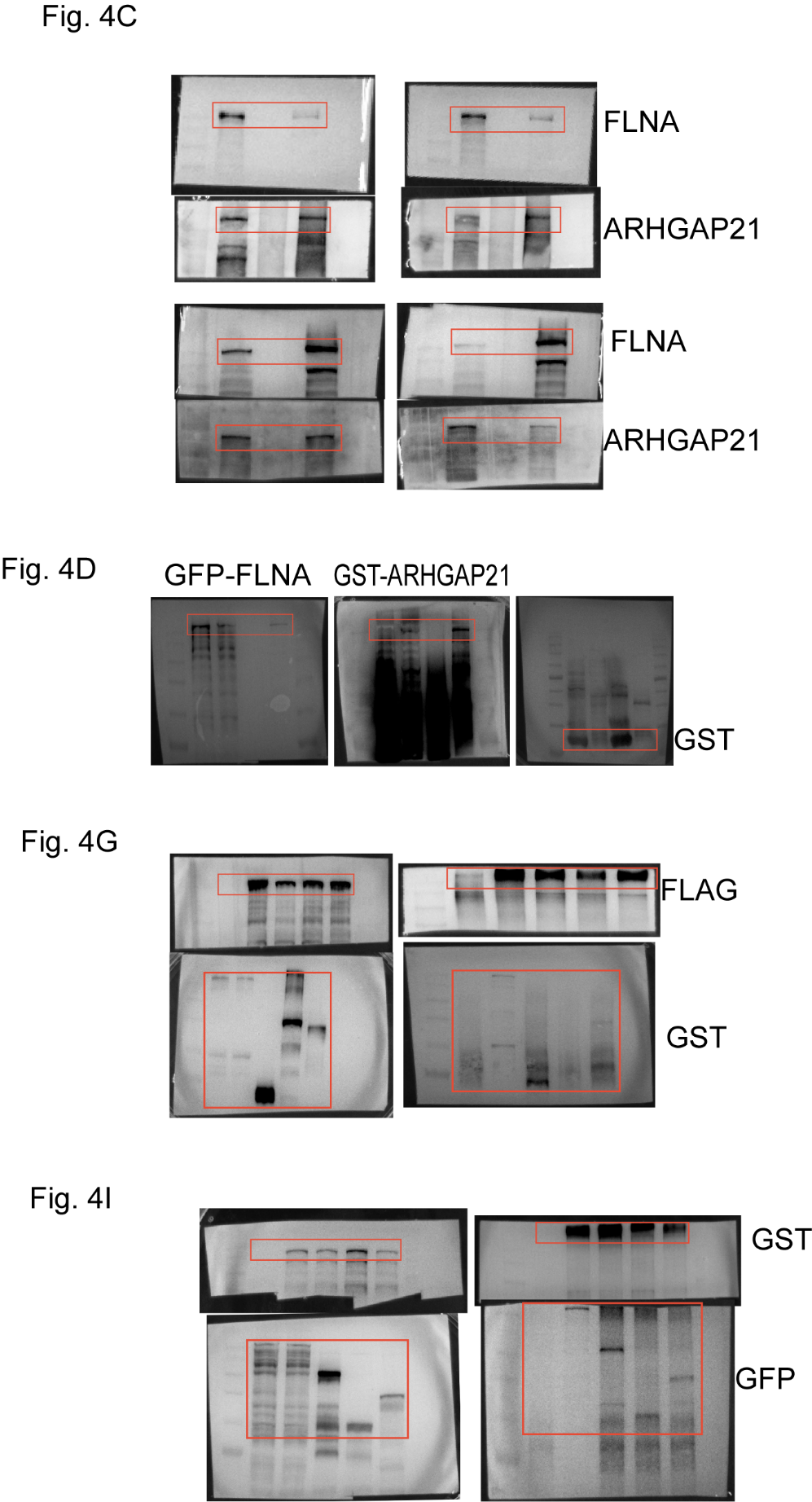


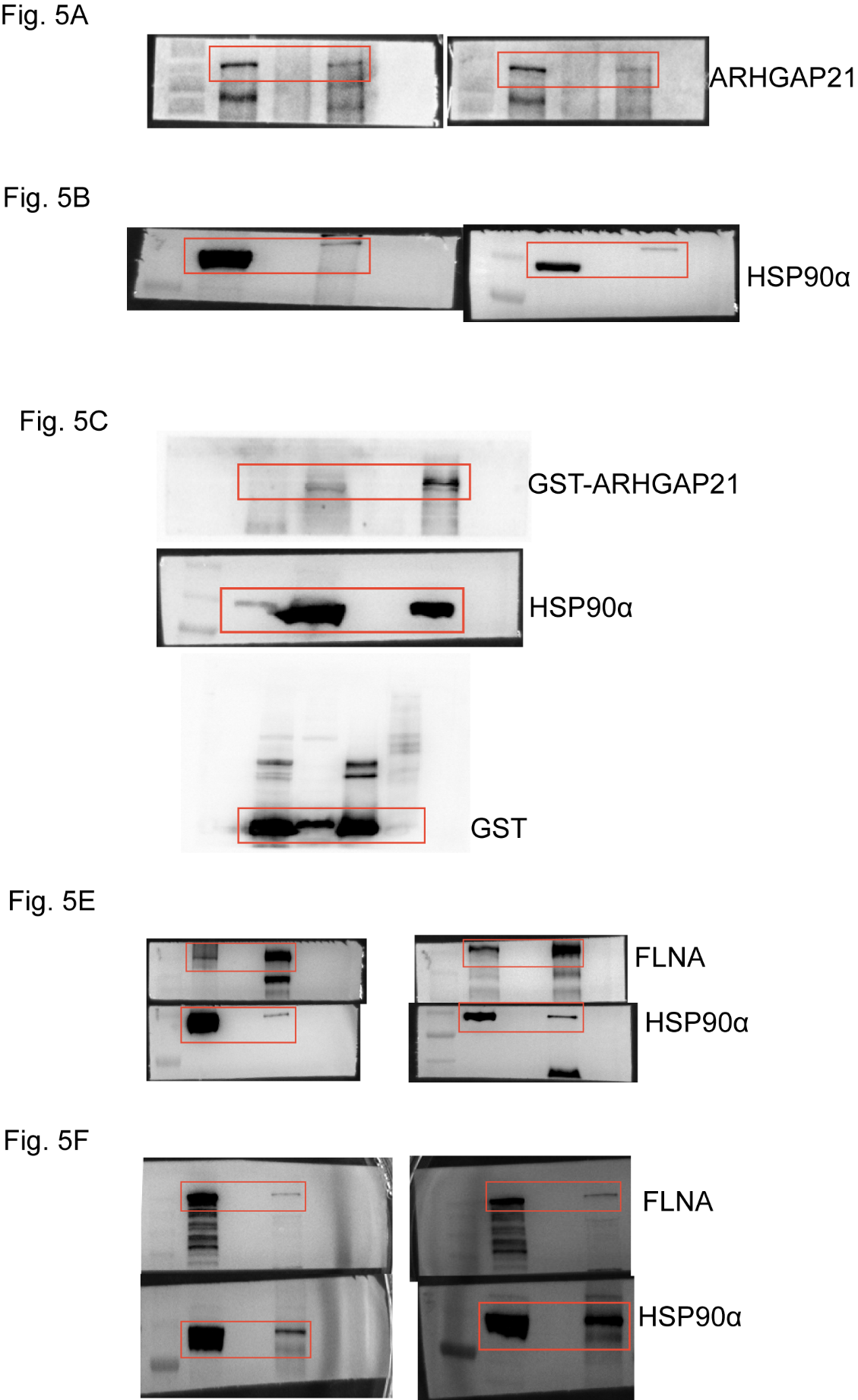


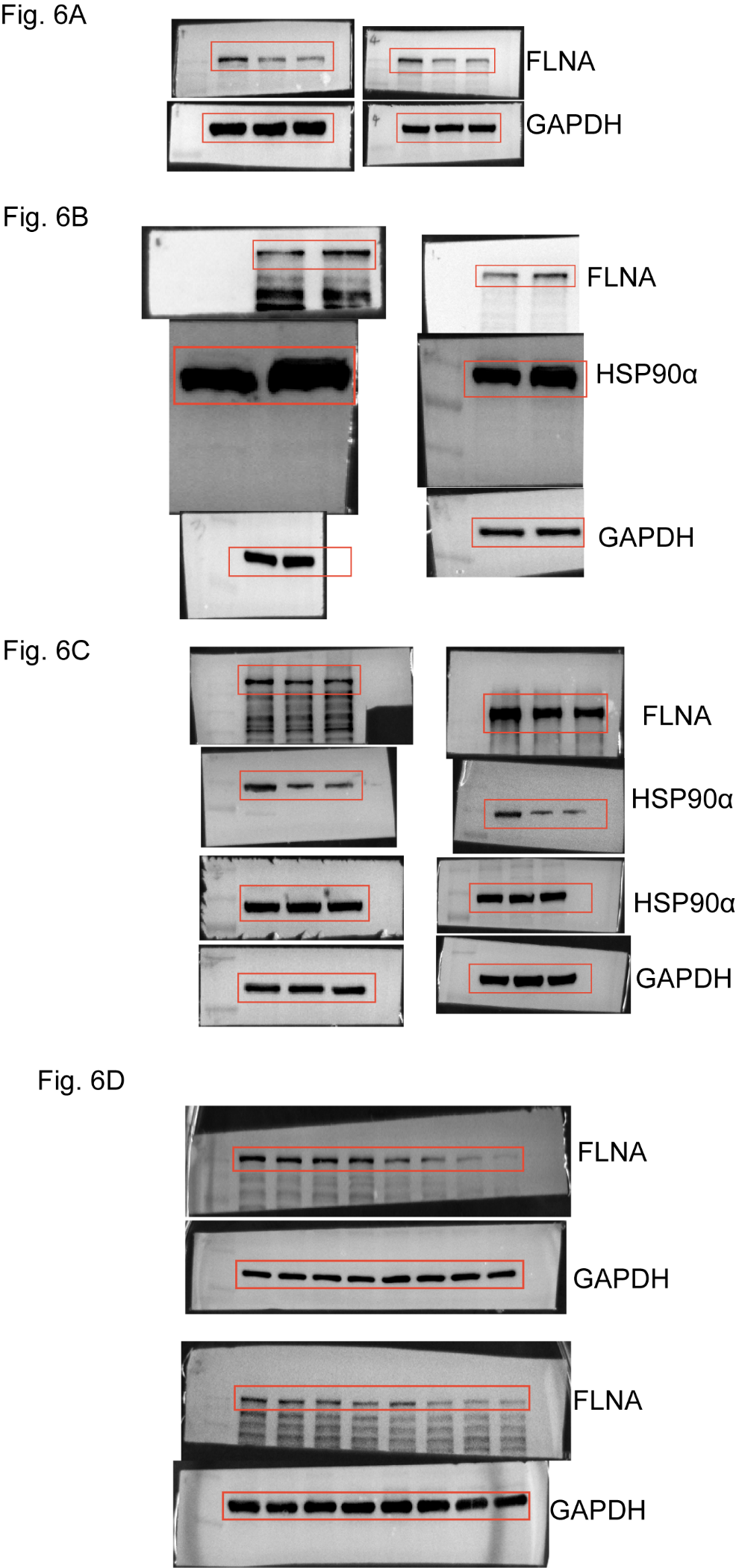


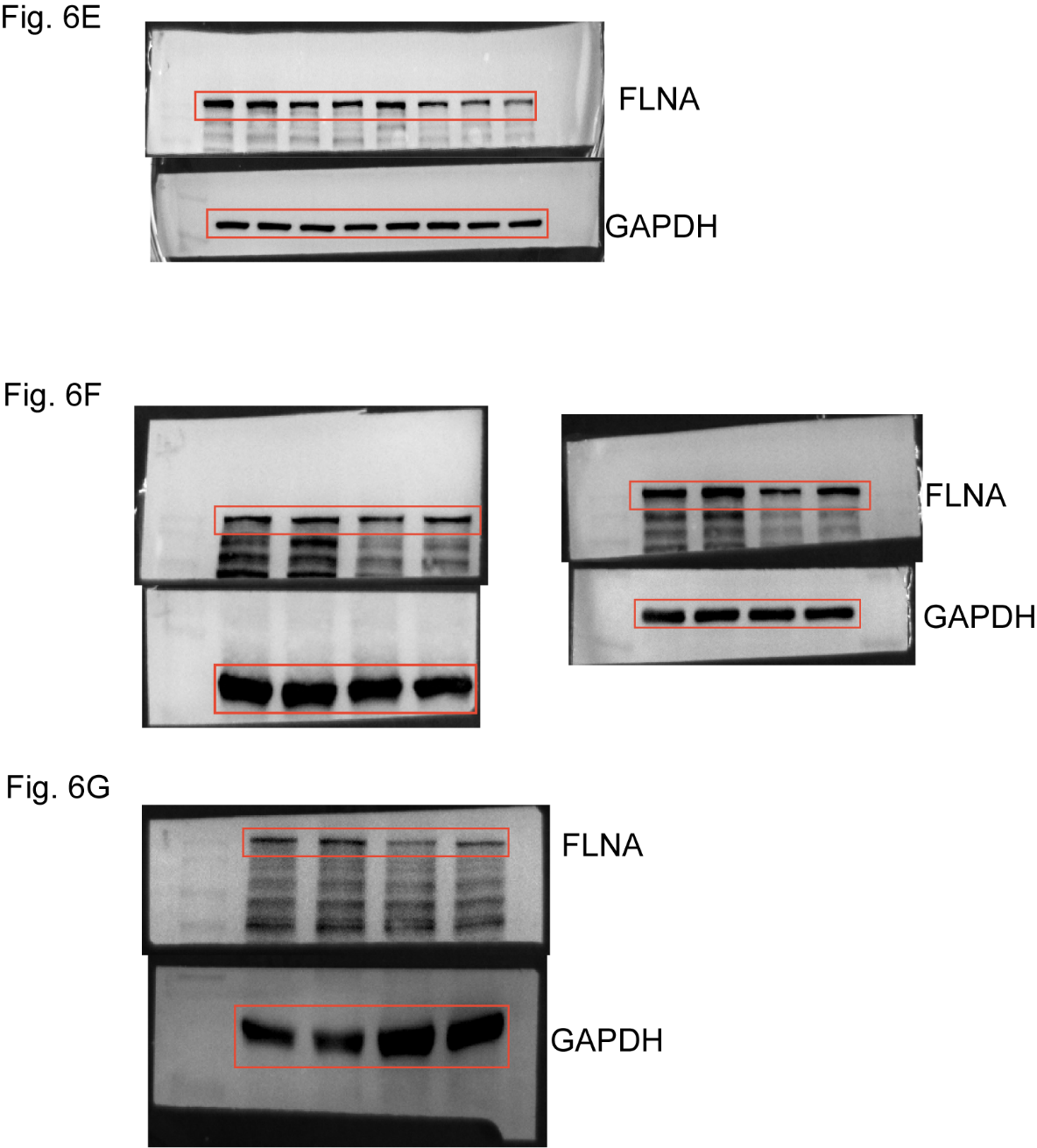


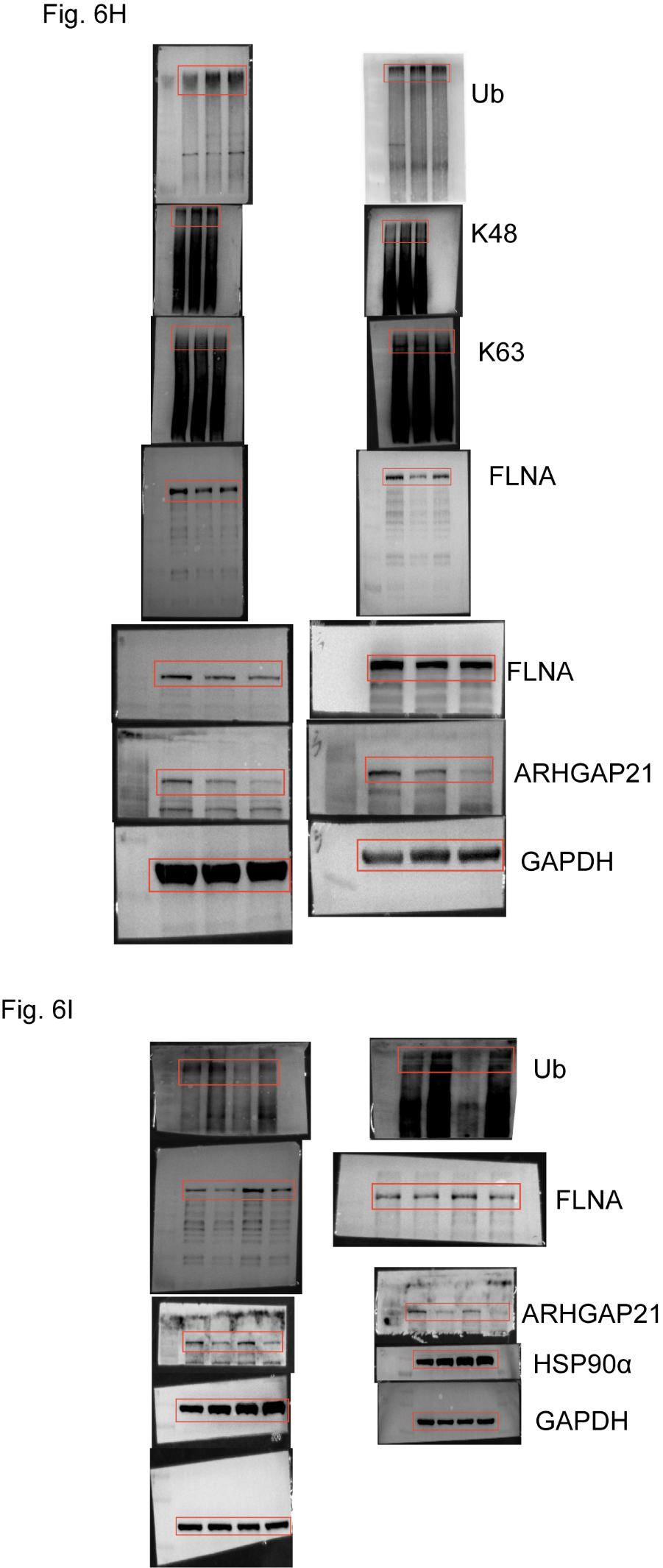


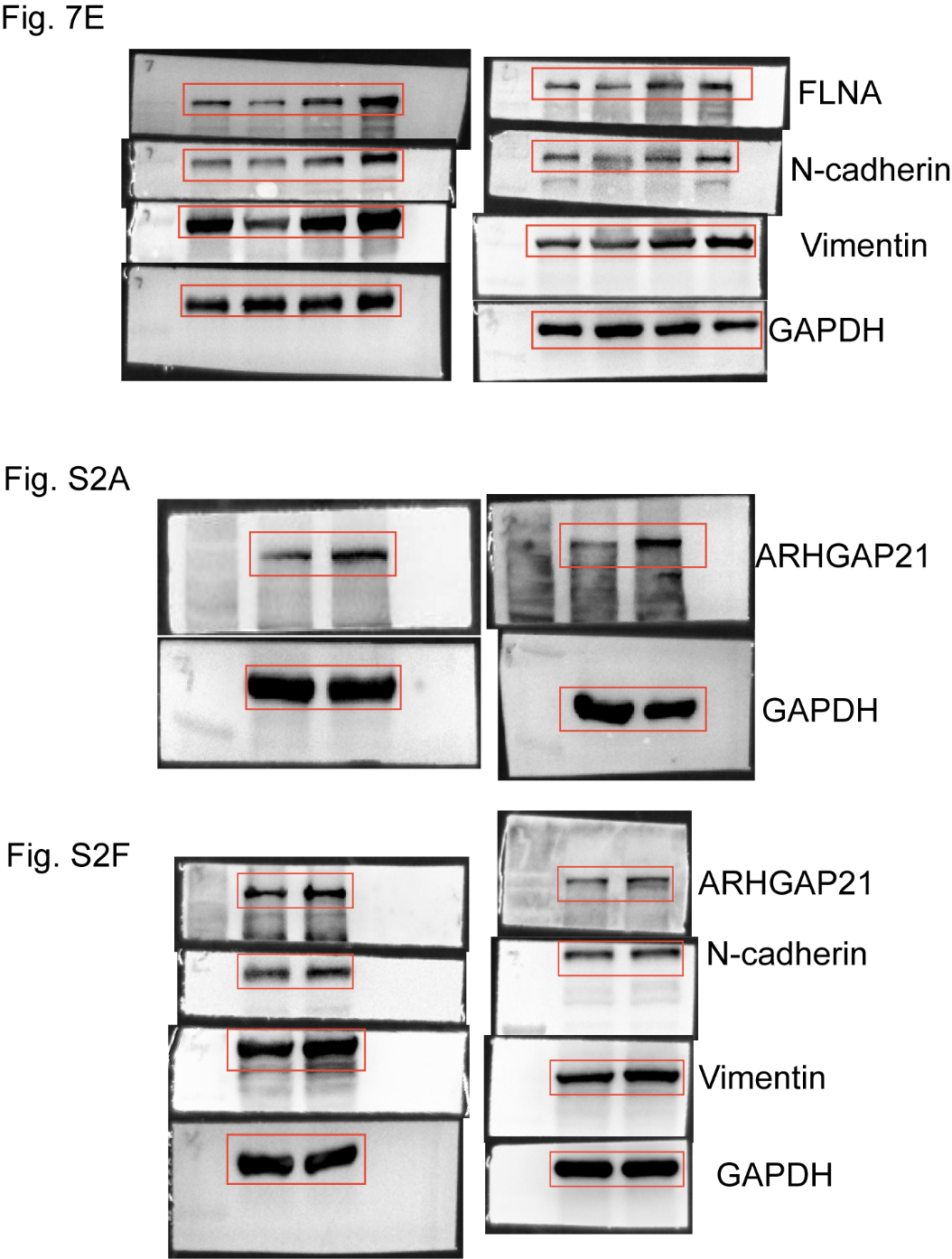

Supplement: Supplementary file 2 — Original Data. [file 41420_2026_3103_MOESM2_ESM.docx]
